# A qualitative analysis of pharmacists’ attitudes towards provision of medication abortion

**DOI:** 10.1186/s12913-023-09543-z

**Published:** 2023-05-30

**Authors:** Selina Sandoval, Grace Chen, Sally Rafie, Borsika Rabin, Sheila Mody, Sarah Averbach

**Affiliations:** 1 Division of Complex Family Planning, Department of Obstetrics, Gynecology and Reproductive Sciences, University of California, 9300 Campus Point Dr. MC 7433, La Jolla, San Diego, CA 92037 USA; 2 School of Medicine, University of California San Diego, San Diego, CA USA; 3 Department of Pharmacy, University of California San Diego Health, San Diego, CA USA; 4 Herbert Wertheim School of Public Health and Human Longevity Science, University of California San Diego, San Diego, CA USA; 5 UC San Diego Altman Clinical and Translational Research Institute Dissemination and Implementation Science Center, University of California San Diego, San Diego, CA USA

**Keywords:** Abortion, Medication abortion, No-test medication abortion, Pharmacist, Pharmacy

## Abstract

**Background:**

90% of United States’ counties do not have a single clinic offering abortion care, and barriers to care disproportionately affect low-income families. Novel models of abortion care delivery, including provision of medication abortion in pharmacies, with pharmacists prescribing medication, have the potential to expand access to abortion care. Pharmacists are well-positioned to independently provide abortion care and are highly accessible to patients, however medication abortion provision by pharmacists is not currently legal or available in the United States. To assess the potential acceptability of pharmacist provision of medication abortion and to identify anticipated barriers and facilitators to this model of care, we explored pharmacists’ attitudes towards providing medication abortion, inclusive of patient selection, counseling, and medication prescribing.

**Methods:**

From May to October 2021, we conducted 20 semi-structured qualitative interviews with pharmacists across the United States, guided by the domains of the Consolidated Framework for Implementation Science Research.

**Results:**

Major themes included there is a need for pharmacist provision of medication abortion and pharmacists perceive provision of medication abortion to be potentially acceptable if anticipated barriers are addressed. Anticipated barriers identified included personal, religious, and political beliefs of pharmacists and lack of space and systems to support the model. Ensuring adequate staffing with pharmacists willing to participate, private space, time for counseling, safe follow-up, training, and reimbursement mechanisms were perceived strategies to facilitate successful implementation.

**Conclusions:**

Pharmacist identified implementation strategies are needed to reduce anticipated barriers to pharmacist provision of medication abortion.

## Background

Approximately 90% of United States’ counties do not have a single clinic offering abortion care, and 39% of women aged 15–44 live within those counties [1]. Travel required to access abortion care leads to increased out-of-pocket costs, including childcare, lodging, food, and lost wages [2]. These barriers disproportionately affect low income families and result in delays of care, further increasing disparities in abortion care and undesired birth rates [2, 3]. Novel models of abortion care delivery, including provision of medication abortion in pharmacies, with pharmacists prescribing medication, have the potential to expand access to abortion care, especially in rural areas [4].

In contrast with facilities offering abortion care, community pharmacies are often more accessible to patients. More than 90% of Americans live within 2 miles of a community pharmacy [5, 6]. Pharmacies commonly have expanded evening and weekend hours. In addition, pharmacists may be well-positioned to provide medication abortion care, as they safely provide other reproductive health care, including hormonal contraception [7].

Medication abortion is approved in the United States by the Food and Drug Administration (FDA) to end a pregnancy through 70 days gestation. The FDA approved regimen includes 200 mg of mifepristone taken orally, followed by 800 mcg of misoprostol taken buccally 24 to 48 h after mifepristone administration [8]. A recent prospective cohort study reported that medication abortion dispensed by pharmacists, following a clinician visit for evaluation and consent, was safe, effective, and acceptable to patients [9]. While pharmacy dispensing of medication abortion regimens, with a prescription from a clinician, has only recently been approved by the FDA in January 2023, pharmacy provision of medication abortion, with pharmacists prescribing medication independently, is not currently offered in the United States, due to current limits in scope of practice [9, 10].

Two trends in abortion care support the potential for pharmacists’ participation in abortion care: the increased utilization of medication abortion among people seeking abortion care and the movement toward “no test” protocols for providing medication abortion care amidst the COVID-19 pandemic [11]. Although the abortion rate in the United States has decreased in recent decades, the proportion of medication abortions has increased from 5% of all abortions in 2001 to 54% of all abortions in 2020 [12]. With the goal of increasing access to medication abortion, especially amidst the COVID-19 pandemic, many clinics have adopted protocols for “no test” medication abortion, which includes guidelines for appropriate patient selection, treatment regimen and follow-up care [11, 13]. A recent nationwide cohort study in the U.K. found that medication abortions provided through telemedicine without ultrasonography or other screening tests have similar safety and efficacy as the traditional in-clinic provision [14]. Additionally, a multicenter retrospective cohort study in the United States found history-based screening for medication abortion to be safe and effective, with outcomes similar to patients undergoing screening ultrasounds and pelvic exams [15].

Using an implementation science lens, we explored the attitudes towards, and potential acceptability of, pharmacists’ provision of medication abortion, inclusive of patient selection, counseling, medication prescribing and follow-up. We identified perceived facilitators and anticipated barriers to implementing pharmacists’ provision of medication abortion.

## Methods

We performed a qualitative cross-sectional study with a non-probability sample of pharmacists. Pharmacists were eligible to participate if they currently prescribed, or were interested in prescribing, hormonal contraception and, therefore, have some familiarity with reproductive healthcare. These pharmacists were identified as possible early adopters of medication abortion provision.

Pharmacists were recruited from across the United States through the Birth Control Pharmacies directory, a United States based directory of pharmacies that self-report offering birth control services [16]. We sought to recruit pharmacists with experience or interest in providing reproductive health services, given their familiarity with anticipated barriers and facilitators to providing reproductive health care. We also aimed to enrich our sample by selecting to interview pharmacists who practiced in multiple settings, including independent community pharmacies, chain community pharmacies, student health pharmacies and health-system pharmacies, and across multiple States. We recruited through an email invitation to participate in the study, sent specifically to pharmacists offering birth control services. Additionally, a recruitment link was posted to a Birth Control Pharmacist social media page. Initially, pharmacists were recruited to participate through convenient sampling, and subsequent invitations to interview were sent to our target participants. We aimed for a sample of 20–25 interviews.

We conducted semi-structured interviews from May to October 2021. The interview guide was designed to explore potential acceptability of medication abortion provision by pharmacists and pharmacists’ attitudes towards providing medication abortion. Interviews were conducted by a single doctorate-level research staff trained in qualitative research methodology and lasted approximately 20–25 min.

We recorded, deidentified, and transcribed interviews using Rev.com [17]. Transcriptions were uploaded to ATLAS.ti version 9.1.3 [18] and we analyzed their content with a directed content analysis approach [19]. Content was sequentially analyzed throughout data collection to refine questions and assess for thematic saturation. Two team members independently reviewed transcripts to identify initial key topics and generate a codebook. These codes were organized into preliminary themes according to the CFIR domains. A third research team member joined to create the master codebook through review of the coded themes in a collaborative process. A subset of data was group coded for consensus-building. After five transcripts independently analyzed by each coder demonstrated 91% inter-coder reliability, the remaining transcripts were each analyzed by one coder. We continued interviews until thematic saturation was reached [20].

**Fig. 1:**
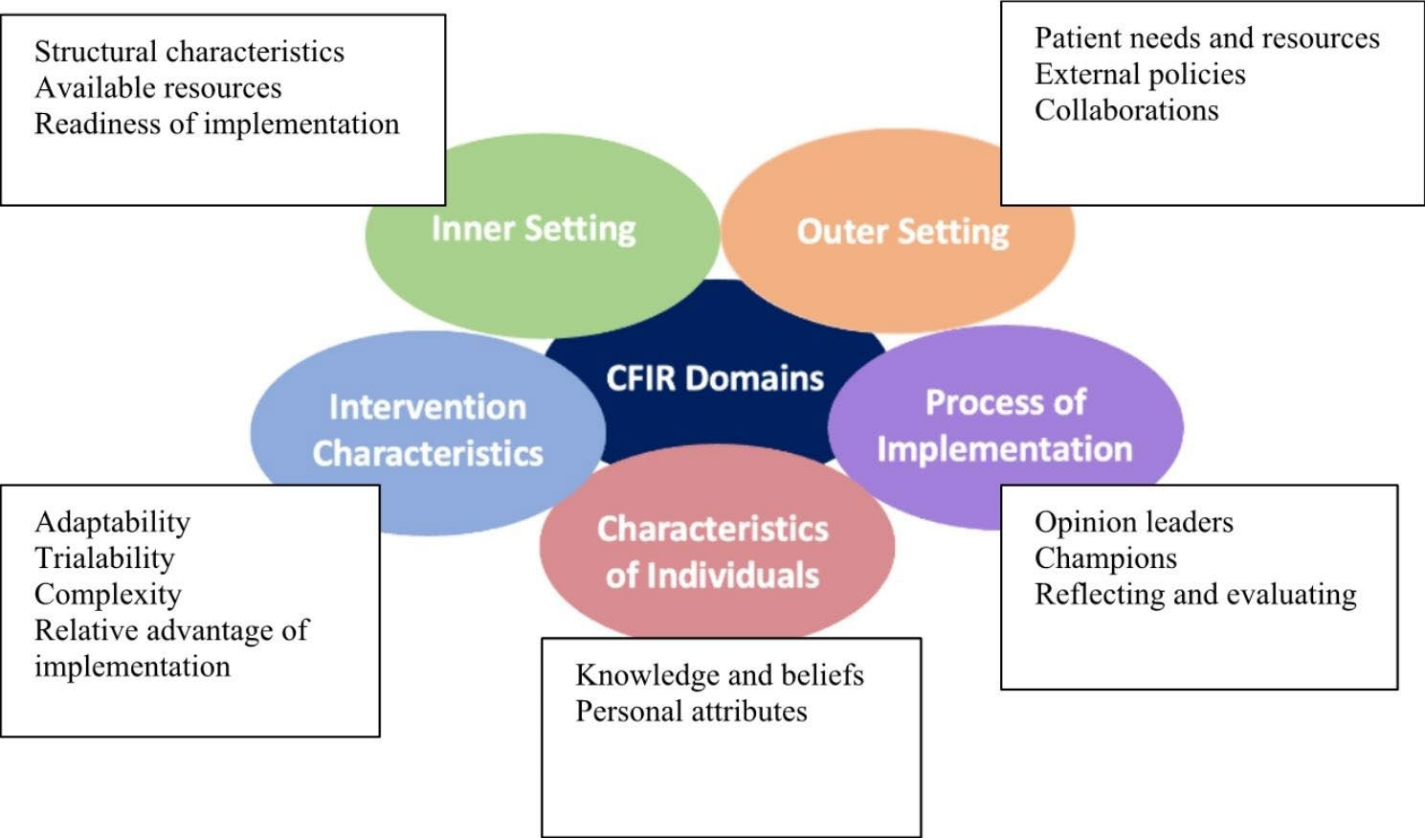
CFIR Domains Figure 1 depicts the five CFIR domains and constructs within each domain for the implementation of pharmacist provision of medication abortion

Implementation Science frameworks support our systematic understanding of key factors that contribute to the implementation of new practices. In this study we used the Consolidated Framework for Implementation Science Research (CFIR) to structure and inform the development of the interview guide and analysis of the interview transcripts [21]. The framework allowed us to explore the pharmacists’ attitudes towards providing medication abortion and identify anticipated barriers and facilitators for the implementation of provision (Fig. 1). Key domains of the CFIR framework include the inner setting, outer setting, intervention characteristics, characteristics of individuals, and the process of implementation. The framework provides a menu of constructs within each domain and offers a practical guide to systematically assessing anticipated barrier and facilitators. The *inner setting* domain addresses the cultural, political, and physical constructs addressing where the abortion provision will take place. Constructs within the inner setting domain include structural characteristics, available resources, and readiness for implementation. The *outer setting* domain includes the social and political contexts of abortion care within which the pharmacies operate. This domain includes the constructs of patient needs and resources, organizational connectiveness (including collaborations), as well as external policies. The *intervention* *characteristics* domain focuses on the specific features of the pharmacist provided medication abortion intervention and how these are perceived by pharmacists. Constructs within the intervention characteristics domain include adaptability, trialability, and complexity of the intervention as well as the relative advantage of implementing an intervention. The *characteristics of individuals* domain includes the mindsets, interests, and priorities of the pharmacists and those within their practices as it relates to pharmacist provided medication abortion. Constructs incorporated within the characteristics of individuals domain include knowledge and beliefs about the intervention and other personal attributes. Finally, *the process of implementation* domain addresses planning, leadership, and execution of the pharmacist provided medical abortion intervention. Constructs within the process of implementation domain include opinion leaders, champions, and reflecting and evaluating [21].

The study protocol was approved by the Human Research Protection Program at our institution. All participants provided written informed consent.

## Results

We conducted 20 interviews with pharmacists from eight states (CA, UT, IL, WI, RI, MD, AZ, and NC); nine participants reported prescribing contraception in their practice at the time of the interview. Eight participants worked in a health-system pharmacy setting, six in an independent community pharmacy, five in a chain community pharmacy, and one in an ambulatory care practice.

Primary themes identified included [1] there is a need for pharmacist provision of medication abortion and [2] pharmacists perceive provision of medication abortion to be potentially acceptable if anticipated barriers are addressed.

### Theme 1: there is a need for pharmacist provision of medication abortion

Pharmacists identified that their unique role as community-based health care providers would facilitate their ability to provide abortion care. Participants recognized the importance of increasing access to abortions, especially in rural counties and states that are hostile to abortion access. Most participants identified the impact pharmacists can make by joining the pool of clinicians providing abortions:

*I think that the state of women’s healthcare and abortion services, especially in the country right now, they need and deserve expanded access. So as a pharmacist, we’ve frequently been considered the most accessible healthcare providers in the entire healthcare system. Where else can you just walk up to a counter and ask a degreed medical professional a question? (Illinois, Health Systems Pharmacy)*

Many participants identified pharmacists as the most accessible member of a healthcare team. They have trusting relationships with patients and could facilitate abortions.

### Theme 2: pharmacists perceive provision of medication abortion to be potentially acceptable if anticipated barriers identified are addressed

#### Potential acceptability

Following review of the “no-test” protocol, most pharmacists felt this model of care was potentially acceptable, and could be incorporated into their current work setting. Pharmacists commonly cited their experience in other areas of health care in their ability to provide medication abortion:

*I think that it would work well… we have a wide scope of practice, we’re adjusting medications for chronic diseases. We have pharmacists involved with oral contraceptives and emergency contraceptives. I think medication abortion can be incorporated to current practices, because if protocols are in place, then it’s something that I think we could adapt to. (California, Health Systems Pharmacy)*

When asked to explain how this work would fit into their current model of care, one pharmacist alluded to the ease of “no-test” medication abortion:

*I think that you could definitely give them more of a questionnaire. There’d be no testing, no ultrasound… you make sure that they’re eligible, counsel them on the medication like we said or the signs and symptoms of anything that they might need to go to the emergency room. (California, Independent Community Pharmacy)*

One pharmacist however raised a concern about receiving accurate information from patients:

*The only concern would be is we don’t know the patient as well, so will they really give us the right information? Will they give us the truth? Will they tell us how it really is? Whereas their OB would know them from the beginning. (California, Chain Community Pharmacy)*

Most participants did not see lack of formal abortion training as a barrier to pharmacist providing medication abortion if adequate training materials were provided and appropriate policies were put into place. Participants expressed that the components of this care could be understood, communicated, and safely provided by their colleagues. One pharmacist reported:

*We go to school for a very long time, learn a lot about medications, pharmacology, physiology, etc. I really don’t think that knowledge is going to be a barrier. (Illinois, Independent Community Pharmacy)*

#### Anticipated barriers and facilitators

We identified anticipated barriers and facilitators to pharmacist provision of medication abortion, which were organized by CFIR domains.

##### Inner setting

(structural characteristics, available resources, and readiness for implementation)

Most participants identified the necessary time required for counseling as a possible barrier to pharmacist provision of medication abortion. This concern was expressed most among participants working in chain community pharmacies but also among participants at independent community pharmacies. Some pharmacists expressed concern over the need to be immediately available to patients requesting medication abortion, which may be difficult depending on the volume of services each day:

*I have a little more time, being a smaller pharmacy, but time might be an issue for a lot of pharmacies, because you don’t want to just quickly push it through without taking a little bit of time to really understanding the patient’s situation. (Utah, Independent Community Pharmacist)*

Another pharmacist proposed established appointment times as a solution to the impact on pharmacist availability. Multiple pharmacists felt they could implement patient abortion visits using similar processes already in place for contraception visits. However, many pharmacists expressed the need for protocols to ensure safe follow up.

*I think the part that might be more complicated and resource intensive is the follow up part. Because they have to check in, you got to make sure they’re not having complications, and then what to do if they are potentially experiencing complications. So that’s the part I’m not 100% clear on yet, but I’m pretty sure we could figure out a way to make it work. (California, Independent Community Pharmacy)*

The “no-test” medication abortion protocol includes follow-up contact with the patient at one week for patient self-assessment of completed abortion and a home high-sensitivity urine pregnancy test 4 weeks from the abortion to confirm a negative result [11]. A few pharmacists shared that it is less common in their practice to arrange follow-up with patients and questioned ways to implement established follow-up contact. Participants reported they would feel greater sense of responsibility over patients as the primary provider of the medications to induce abortion.

Facilitators identified within the Inner Setting domain include access to a private consultation space, adequate time required for comprehensive counseling, and establishing safe follow-up plans. Recognizing the sensitive nature of abortion counseling, many participants highlighted the need for a private space to provide counseling:

*If the stores made some separate rooms or clinic sites, clinical rooms like that, then that would be a little bit more beneficial where the patient would feel like they have the privacy that they need. Because in the pharmacy itself, as much as we try to keep the HIPAA and try to keep privacy, it’s still going to be somebody right next to you. (California, Chain Community Pharmacist)*

Many participants also identified that private consultation spaces would allow patients more comfort to ask questions. Pharmacists felt a private space facilitated the one-on-one attention necessary for counseling and prevented interruptions from other staff or pharmacy patrons. Many pharmacists reporting having access to private space already but some reported they did not have access to private space for consultation.

##### Outer setting

(patient needs and resources, organizational connectiveness)

Within the Outer Setting domain, the main facilitator identified included access to physician support for pharmacist provided medication abortion. Multiple participating pharmacists expressed a desire to have direct access to, and clinical relationships with, clinicians with established practices already providing abortion care as an important facilitator to providing care and meeting patient needs:

*I think pharmacies are resource centers. And I think having resources and partnerships with Planned Parenthood and physicians in the area is important. So, in addition to obviously handouts we would give to patients, I think making the connection between a pharmacist and local providers would be important as well, so creating a support with each other. (California, Independent Community Pharmacy)*

Many participants felt access to clinician (physician and advanced practice clinicians) support would provide additional clinical resources, a place to go directly to ask questions that arise, and a safety structure to refer patients to experienced providers for rare complications.

##### Intervention characteristics

(adaptability, trialability, and complexity of the intervention, relative advantage of implementing an intervention)

Anticipated barriers identified within the Intervention Characteristics domain include the need for adequate training and education on medication abortion and support for pharmacist billing and reimbursement processes. Many participants identified a formal training or credentialing process would facilitate providing high-quality safe abortion care. This process was commonly likened to the required training for vaccine administration or provision of contraception:

*What I can think of is how pharmacists can now prescribe birth control… They would sign up for a class, get trained on all the information, have to maybe do like a competency, and they’re given the certification. And similar with like pharmacist administering vaccines in the community, just having the hands-on experience, and then doing the training. (California, Health Systems Pharmacy)*

One participant proposed offering training that provided Continuing Education credit to those who completed training in medication abortion provision as a strategy to incentivize training.

Providing support for billing and reimbursement for pharmacy services was identified by most participants as a strategy that would facilitate pharmacists provision of abortions. Pharmacists shared that having the time to provide adequate care may be a barrier without a reimbursement mechanism:

*I also would like to look at how to get reimbursed for our time to do this, some type of billing mechanism. I think the pharmacists are used as this person that gives free advice all the time. And it’s great. I mean, we’re providing access to care, which is awesome. But every other healthcare provider gets paid for every minute they talk to a patient. (California, Chain Community Pharmacy)*

Offering compensation for this service was proposed by some pharmacists to facilitate abortion care in pharmacies.

##### Characteristics of individuals

(knowledge and beliefs, other personal attributes)

The most commonly identified barrier within the Characteristics of Individuals domain was the concern for personal, religious, and political beliefs of pharmacists, managers, and other pharmacy staff creating barriers to care. This theme was most prominent amongst pharmacists working in states historically hostile to abortion access:

*Being in a conservative area, I think the difficulty would be finding pharmacists willing to provide the consultation and actually dispense the medication. That’s definitely a little different than California over here, as far as religion and beliefs on that. (Utah, Independent Community Pharmacy)*

In addition, participants shared concerns about pharmacy managers or pharmacy technicians feeling uncomfortable with providing the service within their pharmacy or even blocking access.

##### Process of implementation

(opinion leaders, champions, and reflecting and evaluating)

Within the Process of Implementation domain, pharmacists identified administration support as a facilitator to providing medication abortion in their pharmacies:

*I think trying to get buy-in and commitment from the organizations in which these pharmacists are working to facilitate a different practice model to allow pharmacist time to collect the data they need from the patient, make sure that that patient meets screening criteria …and then that they can safely be provided the medication. (Wisconsin, Independent Community Pharmacy)*

A few pharmacists identified administration support as a tool to implement policies and protocols for pharmacists to provide medication abortion. Support from pharmacy administration was also seen as a tool to combat other barriers, such as personal objections to abortion care.

## Discussion

We found that most participating pharmacists perceive that there is a need for pharmacist provision of medication abortion and pharmacist provision of medication abortion is potentially acceptable if the anticipated barriers identified are addressed. Provision of medication abortion by pharmacists could lead to increased access to medication abortion in the United States, especially in rural areas. Our findings highlight that there are key barriers that need to be addressed to support successful implementation. These include lack of connection between pharmacies and clinicians already experienced in providing abortion services, lack of knowledge and experience in abortion provision, and lack of systems for billing and reimbursement. Additional data are needed prior to successful implantation at scale. For example, a pilot study can be used to assess the implementation strategies required for pharmacists to successfully provide medication abortion. This can be followed by an implementation-effectiveness trial to assess for safety, effectiveness, and implementation outcomes. Currently, state policies allow for independent pharmacist provision of contraception in 9 states, with an additional 10 states allowing pharmacist contraception prescribing under standing orders or collaborative practice agreements [22]. However, pharmacist provision of medication abortion is not permitted in any state due to current limits in scope of practice, and the FDA only recently approved registered pharmacies dispensing mifepristone directly to patients with a prescription from a clinician [10]. Additionally, pharmacists would need to be able to be registered as mifepristone prescriber as required by the Food and Drug Administration’s Risk Evaluation and Mitigation Strategy (REMS).

Strategies to support implementation of pharmacist provision of medication abortion were identified that would lead to increased interest in offering this service. First, a system to support follow-up is needed, especially in the event of a rare complication. Second, formal partnerships between pharmacists and clinicians providing abortion services or post-abortion care can support expansion of safe abortion services and facilitate expedited access to experienced providers for consultation and transfer of care when appropriate.

A third strategy included forming structured training curriculums with opportunities for continuing education credit. Pharmacists suggested that a formal training program would address concern for liability and patient safety considerations. This training could be provided through online modules and/or in-person classes, to allow for discussion and offer the opportunity for questions.

A fourth strategy identified involved creating systems for billing and reimbursement to facilitate the implementation of pharmacist provision of medication abortion. Support should include formal training of pharmacists and pharmacy leadership.

Each of the suggested strategies above require the prioritization, co-creation, and piloting of formally operationalized implementation strategies prior to full implementation of this model of care. The barriers and facilitators identified in our study are similar to those identified in a study using semi-structured interviews to explore pharmacist experiences with *dispensing* abortion medications in Canada, following clinician prescription. These included time and resources, expert and peer opinions, champions and external collaboration [23]. Additionally, the findings of our study are similar to those from in-depth interviews of pharmacy owners and midwives in Nepal where participants felt they could safely provide medication abortion and fill an important role in convenient and confidential provision of care in their communities [24]. A non-inferiority study in Nepal evaluated medication abortion provided by trained midwives in pharmacies versus in a clinic based settings and found no difference in efficacy between the settings [25, 26]. Similarly, pharmacy dispensing of medication abortion in Australia has led to improved access to medication abortion, specifically in rural areas [27].

Concern about personal and religious beliefs was a commonly anticipated barrier. These concerns were addressed in other health care settings through values clarification workshops. These workshops commonly lead to improvement in abortion knowledge and attitudes, which are often rooted in abortion stigma. [28] We acknowledge that abortion is a complex issue for some health care members, but feel the focus on the autonomy, health, and well-being of our patients can serve to improve patient experience and access.

There are limitations of this study, including the sampling which intentionally selected for pharmacists interested in reproductive health to enrich our understanding of potential facilitators but may bias the responses towards those who feel this model of care is potentially acceptable. These findings may not be generalizable throughout the United States, especially in states hostile toward reproductive health care. However, this research will begin to provide preliminary data about a novel model of medication abortion provision that has potential to greatly increase access nation-wide.

Our study has several strengths. We utilized an implementation science framework, CFIR, to guide our study design and analysis, allowing for our findings to support implementation. Our study is novel in evaluating the attitudes of pharmacists’ about provision of medication abortion in the United States. Although pharmacist dispensing of medication abortion was studied and found to be both effective and acceptable to patients, little is known about pharmacists’ attitudes towards medication abortion provision and their willingness to provide this service in the U.S [9]. Whether this model would be feasible and acceptable to pharmacists and patients warrants further investigation. Patients trust pharmacists in other aspects of their reproductive health care, including provision of hormonal contraception and emergency contraception, but we have not evaluated their attitudes towards pharmacist provision of medication abortion.

## Conclusions

Our data suggests pharmacists perceive provision of medication abortion as potentially acceptable, however they identified anticipated barriers which must be addressed for successful implementation at scale. We recommend a pilot study testing implementation strategies and implementation toolkit development. Additional data has the potential to support changes in the legal framework to allow pharmacists to provide medication abortion in the United States.

The political climate around abortion in the United States requires that we continue to explore alternative methods to provide abortions. New models have the potential to impact abortion access. With further investigation into this model of abortion care, pharmacist provision of medication abortion has the potential to increase abortion access, especially for patients in rural areas. Increasing access in rural counties increases equity in abortion access, allowing for faster presentation to care and ease of obtaining services.

## Data Availability

The datasets used and analyzed during this study are available from the corresponding author on reasonable request.

## List of Abbreviations

CFIR: Consolidated Framework for Implementation Science Research
